# Mapping choline metabolites in normal and transformed cells

**DOI:** 10.1007/s11306-020-01749-0

**Published:** 2020-11-29

**Authors:** Irena Roci, Jeramie D. Watrous, Kim A. Lagerborg, Mohit Jain, Roland Nilsson

**Affiliations:** 1 Cardiovascular Medicine Unit, Department of Medicine, Karolinska Institutet, 171 76 Stockholm, Sweden; 2 Division of Cardiovascular Medicine, Karolinska University Hospital, 171 76 Stockholm, Sweden; 3 Center for Molecular Medicine, Karolinska Institutet, 171 76 Stockholm, Sweden; 4 Department of Medicine & Pharmacology, University of California, San Diego, 9500 Gilman Avenue,, La Jolla, CA 92093 USA

**Keywords:** 13C3 choline, Betaine, CHDH, Methylation, Isotope tracing

## Abstract

**Introduction:**

Choline is an essential human nutrient that is particular important for proliferating cells, and altered choline metabolism has been associated with cancer transformation. Yet, the various metabolic fates of choline in proliferating cells have not been investigated systematically.

**Objectives:**

This study aims to map the metabolic products of choline in normal and cancerous proliferating cells.

**Methods:**

We performed ^13^C-choline tracing followed by liquid chromatography-high resolution mass spectrometry (LC-HRMS) analysis of metabolic products in normal and in vitro-transformed (tumor-forming) epithelial cells, and also in tumor-derived cancer cell lines. Selected metabolites were quantified by internal standards.

**Results:**

Untargeted analysis revealed 121 LCMS peaks that were ^13^C-labeled from choline, including various phospholipid species, but also previously unknown products such as monomethyl- and dimethyl-ethanolamines. Interestingly, we observed formation of betaine from choline specifically in tumor-derived cells. Expression of choline dehydrogenase (CHDH), which catalyzes the first step of betaine synthesis, correlated with betaine synthesis across the cell lines studied. RNAi silencing of CHDH did not affect cell proliferation, although we observed an increased fraction of G_2_M phase cells with some RNAi sequences, suggesting that CHDH and its product betaine may play a role in cell cycle progression. Betaine cell concentration was around 10 µM, arguing against an osmotic function, and was not used as a methyl donor. The function of betaine in these tumor-derived cells is presently unknown.

**Conclusion:**

This study identifies novel metabolites of choline in cancer and normal cell lines, and reveals altered choline metabolism in cancer cells.

**Electronic supplementary material:**

The online version of this article (10.1007/s11306-020-01749-0) contains supplementary material, which is available to authorized users.

## Introduction

**Fig. 1:**
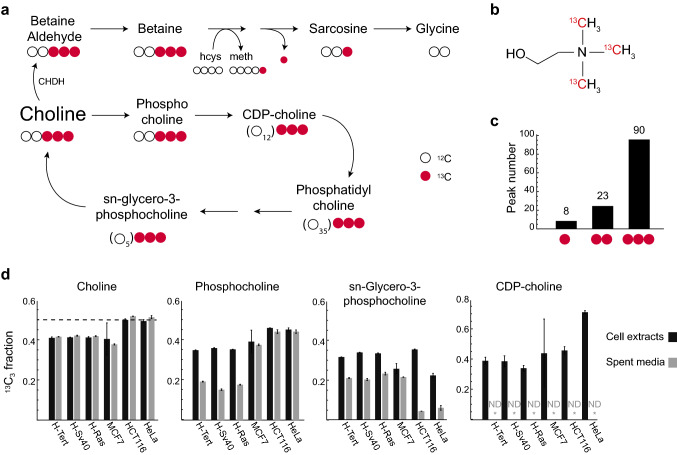
**a** Schematic of choline metabolism and ^13^C_3_-choline tracing. **b** The structure of ^13^C_3_-choline used for tracing experiments. **c** number of LC-HRMS peaks that acquired 1, 2 or 3 ^13^C from ^13^C_3_-choline. **d** ^13^C_3_ mass isotopomer fraction of choline and related metabolites in the cell extracts and spent media of different cell lines. Dotted line indicates the expected fraction of choline labeling in fresh culture medium (50%)

Choline is a central nutrient in human metabolism. Choline forms the head group of phosphatidylcholine, the most abundant lipid in cell membranes (van der Veen et al. 2017), and choline is therefore required in substantial amounts by proliferating cells to synthesize membranes. Although choline can be synthesized from ethanolamine, this occurs mainly in the liver (Vance and Ridgway 1988), and for most proliferating cell types, choline is considered essential. It is present in virtually all cell culture media at substantial concentrations, usually around 10–100 µM, which is comparable to most amino acids. In the liver, choline is also an important source of methyl groups: in this case, choline is oxidized to betaine, which donates one methyl group to the methionine cycle in a reaction catalyzed by betaine-homocysteine methyl transferase (BHMT), thus supporting a variety of methylation reactions (Fig. 1A). The BHMT reaction yields dimethylglycine, containing the remaining two methyl groups, which can then be donated to the folate-carried one-carbon pool. BHMT expression is restricted to liver and kidney (McKeever et al. 2015), while in proliferating cells, methionine and serine are considered the main sources of one-carbon groups (Tibbetts and Appling 2010).

In cancer, elevated choline metabolites in the phosphatidylcholine synthesis pathway have long been observed (Daly et al. 1987). Transformation of epithelial cells increases the abundance of these metabolites independent of proliferation rate, suggesting that the enzymes of phosphatidylcholine synthesis are under control of oncogenic signaling (Aboagye and Bhujwalla 1999). The most studied enzyme is probably choline kinase (CHKA), the initial step of this pathway, which is a target of the hypoxia-inducible factor (HIF)-1a (Glunde et al. 2008), stimulated by oncogenic signals, and overexpressed in several tumor types (Glunde, Bhujwalla, and Ronen 2011).

Although there is a large body of work characterizing the various enzymes of choline lipid metabolism in cancer, to our knowledge there has been no systematic analysis of choline metabolism in either normal or transformed proliferating cells. Here, we present an unbiased study of possible fates of choline using ^13^C choline tracing. We report evidence for previously uncharacterized products of choline, as well as a curious phenomenon of betaine synthesis in tumor-derived cells, the purpose of which is still unknown.

## Materials and methods

### Cell culture and stable isotope tracing

HCT116 (human colorectal cancer) (ATCC, CCL-247), MCF7 (human breast adenocarcinoma) (ATCC, HTB-22) and HeLa (human cervix adenocarcinoma) (ECACC, 93021013) were maintained in RPMI-1640 (Gibco, 61870–010) + 5% FBS (Gibco, 16140–071) + 1% Pen-Strep (Gibco, 15140–122) for 96 h, whereas HMEC (human mammary epithelial cells hTert immortalized) (Elenbaas et al. 2001), HMEC SV40 (expressing SV40 large-T antigen) (Elenbaas et al. 2001) and HMEC Ras (H-*ras*V12 transformed) (Elenbaas et al. 2001) were maintained in MDCB170 medium (USBiological, M2162) + 5% MEGS (Invitrogen, S-015–5) for 72 h. Cells were seeded at a density of 0.2*10^6^ per well in a 6-well plate.

For choline tracing experiments, 3 mg/L ^13^C_3_-Choline Chloride (IsoSciences, EFL6-2015-217A1) was added to the culture medium. The choline amount in the unlabeled culture medium (provided by vitamin mix) was also 3 mg/L, making the total choline in the labeled medium 6 mg/L (~ 60 µM), of which ~ 50% is ^13^C_3_ choline.

Experiments were performed in duplicates for unlabeled samples and triplicates for ^13^C_3_-Choline labeled samples.

### Metabolite extraction

For metabolite extraction of cells in the culture dish, culture media was aspirated, cells were rinsed with PBS, and the culture dish was placed on a cold plate. 500 µL of ice cold methanol (VWR, BAKR8402.2500) was added to each well for quenching and metabolite extraction, cells were scraped, transferred to a new tube, and kept in -80 °C until further analysis.

Besides cell extracts, a fraction of spent media, fresh media, and culture media incubated at 37 °C in 6-well plates for 96 h were collected for metabolite analysis. Liquid Chromatography – High Resolution Mass spectrometry (LC-HRMS) analysis of samples was performed as described in (Roci et al. 2019).

### LC-HRMS data processing

#### Targeted analysis

LC-HRMS chromatograms were retrieved from samples using a data access layer, mzAccess (Lyutvinskiy et al. 2017), and processed using Wolfram Mathematica (Wolfram Research). Choline, betaine, betaine aldehyde, phosphocholine and sn-glycero-3-phosphocholine were identified based on m/z and retention time (rt) data previously verified by standards on the same method, and the respective mass chromatograms were extracted.

#### Untargeted analysis

Untargeted analysis was performed for the 12 unlabeled cell extract samples from 6 cell lines using an in-house developed peak detection software. Detected peaks were required to have an apex intensity of at least 500,000, be present in at least 3 out of 12 samples, but not present in blanks. The obtained m/z and rt data were used to analyzed the he putative metabolite peaks in a targeted way in the ^13^C_3_-choline labeled samples. Mass isotopomer distributions (MIDs) were calculated for each metabolite peak, and those that were labeled in at least one of the cell lines were selected for further analysis. The peaks were curated manually to select only good quality peaks while avoiding potential noise or false peaks.

The putative metabolite peaks were annotated by matching each peak to entries from the Human Metabolome Database (HMDB) (Wishart et al. 2013) based on the estimated m/z, within 10 ppm accuracy and accounting for + H and –H adducts. Other annotations were retrieved from matching collected MS^2^ spectra against the publicly available library GNPS online tool (Wang et al. 2016). The putative metabolite annotations, m/z, rt, HMDB annotations, GNPS annotations are available in Table S1.

All primary LC-HRMS data will be publicly available via the authors’ website. mzAccess is publicly available at mzaccess.org.

### Metabolite quantification and concentration estimation

For quantification of choline-related metabolites, a mix of pure standards of choline (Sigma-Aldrich, C7527), betaine (Sigma-Aldrich, 61962) and phosphocholine (Sigma-Aldrich, P0378) at known concentrations was added to ^13^C-choline-labeled cell extracts, and to the corresponding spent and fresh medium samples.

Concentration estimation for choline, betaine and phosphocholine was done based on the observed MIDs in labeled cell extracts with and without added standards, and the known concentration of standards added to the labeled sample. The formula used is

$$\left( {{\text{C}}_{{{\text{LA}}}} *{\text{ X}}_{{{\text{LA}}}} } \right)\; + \;\left( {{\text{C}}_{{{\text{STD}}}} *{\text{ X}}_{{{\text{STD}}}} } \right)\; = \;\left( {{\text{C}}_{{{\text{LA}}}} \; + \;{\text{C}}_{{{\text{STD}}}} } \right)\;*\;{\text{X}}_{{{\text{MIX}}}}$$

where C is concentration and X is the ^13^C_3_ mass isotopomer fraction. X_MIX_ is the mass isotopomer of each metabolite in the labeled samples that contain standard mix, C_STD_ is the known concentration of standard added to the labeled sample. C_LA_ is the unknown concentration of the metabolite in the labeled sample which was found by solving this equation while other variables are known.

### Transient knockdown

Knockdown of choline dehydrogenase (CHDH) was performed in HeLa, MCF7 and HCT116 cell lines using 7 different siRNAs from 2 different vendors: CHDH (55349) siRNA Oligo Duplex (Origene, SR310700) and ON-TARGETplus CHDH (55349) siRNA (Dharmacon, LQ-008123-01-0002). For Dharmacon siRNA ON-TARGETplus, Non-targeting Pool (Dharmacon, D-001810-10-05) was used as a negative control.

70,000 cells were seeded in each well in a 6-well plate and cultured for 24 h. The next day, medium was replaced and transfection mixture containing each of the siRNAs was added dropwise into each well, respectively. The transfection mixture contained 200 µL Optimem (Thermo Fischer, 31985–062), a volume of 2 mM siRNA solution and 4µL Interferin (Polyplus, 409–10) per well. The volume of siRNA added was calculated to achieve the final concentration of siRNA indicated in the text. The experiment was ended after 70 h.

For choline tracing experiments in CHDH knockdown cells, cells were cultured and transfected as described above, with the difference that at the day of transfection, medium was replaced with fresh RPMI containing 50% ^13^C_3_-choline. At the end of the transfection experiment, the culture medium was aspirated, cells were rinsed with PBS and treated with warm trypsin (Gibco, 25300–062) for 5 min at 37 °C. Then culture medium was added to deactivate trypsin, and cells were centrifuged at 1500 rpm. Cells were re-suspended in 0.5 mL PBS, and 10 µL of the cell suspension was used to count cells using a Bio-Rad automated cell counter TC20. Cells were then centrifuged again, the pellet was re-suspended in 5 µL, and 500 µL methanol was immediately added to quench and extract metabolites. Extracts were transferred to a new Eppendorf tube, and kept in -80 °C until LCMS analysis.

### Western blot

CHDH protein expression was characterized using the western blot technique. First cells were cultured as described above, rinsed with PBS, and lysed in RIPA buffer (Pierce, 89900) containing protease and phosphatase inhibitor (Thermo Fischer, 78440). The cell lysates were kept in agitation at 4 °C, then centrifuged for 10 min at 13,000 g at 4 °C, and the supernatants were finally transferred to a new tube. 10 μL of the supernatant was used for a BCA assay to measure total protein content, while the remainder was mixed with loading buffer, and incubated at 95 °C for 5 min. 10 µg of each sample was loaded onto a PAGE gel which was run at 100 mV for around 40 min. Meanwhile, PVDF membrane was immersed in methanol for 1 min, and filter papers were soaked in the transfer buffer (Thermo fischer, 84731) for 5 min. After electrophoresis, the gel was blotted using a Thermo Scientific Pierce G2 Fast Blotter, according to manufacturer instructions. The membrane was incubated in 5% milk + PBST (PBS + 0.5% Tween20) for 1 h at room temperature, then incubated overnight with primary antibody at 1:500 dilution in 5% milk + PBST at 4 °C, washed with PBST 3 times, and finally incubated with secondary antibody at 1:5000 dilution in 5% milk + PBST at 4 °C. The membrane was washed 3 times with PBST, incubated with developing reagents (Thermo Fisher, 34096), and imaged using a Vilber Lourmat Fusion Solo chemo luminescence camera. Developed membranes were incubated for 15 min with Coomassie (Bio-Rad, 1610436) to measure total protein content, rinsed in H_2_O for ~ 2 h, dried and visualized in Vilber Lourmat Fusion. CHDH (C-5) mouse primary antibody (Santa Cruz, sc-393885) and goat anti-mouse IgG1-HRP secondary antibody (Santa Cruz, sc-2060) were used for detection of CHDH expression.

## Results

### ^***13***^***C-choline tracing***

While choline is considered an essential nutrient for proliferating cells, and is provided in relatively high concentrations in culture media, there is little data on the metabolic fates of choline in proliferating cells. We chose to study a normal, telomerase-immortalized human mammary epithelial cell (HMEC-Tert) and two derived cell lines that were stepwise transformed with the SV40 early region proteins (HMEC-SV40) followed by a mutant Ras protein (HMEC-RAS), where the latter is tumorigenic in vivo (Elenbaas et al. 2001); and also the tumor-derived cell lines MCF7 (breast carcinoma), HCT116 (colon carcinoma) and HeLa (ovarian cancer). To identify the fate of choline in these cells, we performed isotope tracing with a ^13^C_3_-choline tracer with ^13^C methyl groups (Fig. 1a, 1b). We cultured each cell line with 50% ^13^C_3_-choline for 96 h and then performed untargeted, liquid chromatography-high resolution mass spectrometry (LC-HRMS) analysis of cell extracts. This resulted in a total of 4,908 LC-HRMS peaks, of which 121 were ^13^C-labeled in at least one of the cell lines studied, and thus represent metabolites formed from choline (Table S1). Of these, 90 (74%) were ^13^C_3_, indicating that they contained an intact –N(^13^CH_3_)_3_ head group (Fig. 1c), including phosphocholine and CDP-choline, which are intermediates in phospholipid synthesis; sn-glycero-3-phosphocholine, which is produced by phospholipid catabolism; and also betaine (Fig. 1a, 1d). Phosphocholine and sn-glycero-3-phosphocholine were also found in the spent, but not fresh medium (Fig. 1d). However, some sn-glycero-3-phosphocholine seems to be produced when incubating the serum containing medium, indicating that it can be produced spontaneously from serum lipoprotein. Many other ^13^C_3_ peaks were likely phospholipids (Table S1), showing that phospholipid synthesis from choline occurs in these cells.

### Choline does not contribute to methylation, but is catabolized

**Fig. 2:**
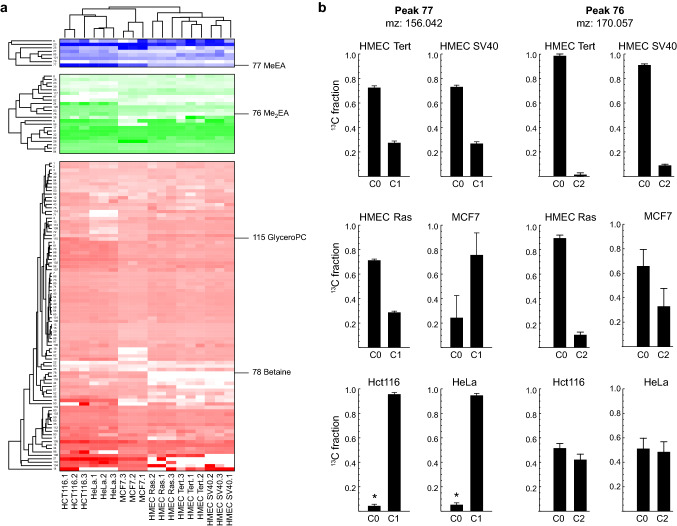
**a** Heatmap of the ^13^C_1_ (blue), ^13^C_2_ (green) and ^13^C_3_ (red) mass isotopomer fraction for 121 LC-HRMS peaks in HMEC Tert, HMEC SV40, HMEC Ras, HeLa, HCT116 and MCF7 cells. **b** Mass isotopomers quantified for peak 77 (mz = 156.0419), putatively annotated as N-Methylethanolaminium phosphate (MeEA-P), and peak 76 (mz = 170.057), annotated as dimethylethanolamine (Me_2_EA-P). Asterix (*) denotes likely underestimated MI fractions due to weak LCMS signal

Besides the various products containing the intact choline head group, we observed 7 and 18 peaks with ^13^C_1_ and ^13^C_2_ mass isotopomers, respectively (Fig. 1c), indicating products that contain only 1 or 2 of the choline methyl groups. The presence of these mass isotopomers across the various cell lines are shown in Fig. 2a. Yet, we found no ^13^C incorporation into methionine, SAM, or known methylated species like nicotinamide in any of the cell lines, showing that choline did not contribute to SAM-dependent methylation in any of the cell lines (Fig. S1). Hence, these cell lines must obtain methyl groups entirely from methionine or folate-carried one carbon units. Furthermore, this implies that the observed ^13^C_2_ and ^13^C_1_ species are likely catabolites of choline, rather than methylation products. While the identity of most of these features are not known, putative identities can be predicted from exact mass and the ^13^C labeling pattern. For example, we noted one peak at m/z = 156.042 with a clear ^13^C_1_ MI, and one at m/z = 170.057 with a ^13^C_2_ MI, which may represent M + H adducts of ^3^C_1_ methyl ethanolamine phosphate (MeEA-P) and ^13^C_2_ dimethyl ethanolamine phosphate (Me_2_EA-P), respectively (Fig. 2b). These features were not in-source fragments of phosphocholine, since their elution profiles clearly differed. Methyl- and dimethyl EA compounds are intermediates in the synthesis of phosphatidyl(Ptd)-choline from Ptd-EA by the PEMT enzyme, but this pathway would not yield the observed ^13^C labeled species when operating in the synthesis direction. Our data instead suggests that MeA-P and Me_2_EA-P can be formed from choline. The enzyme(s) responsible for this activity is not known.

### Betaine is formed by CHDH in tumor-derived cells, but is not used for methylation

**Fig. 3:**
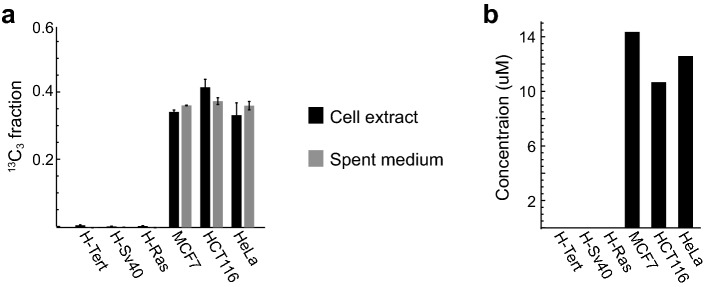
**a** ^13^C_3_ mass isotopomer fraction of betaine in cell extracts and spent media of different cell lines. **b** concentration of betaine in cell extracts of selected cell lines

Interestingly, betaine was synthesized from choline in the tumor-derived HCT116, HeLa, and MCF7 cells, but not in any of the HMEC lines (Fig. 3a). While betaine is a major methyl donor in the liver (Finkelstein and Martin 1984), the lack of choline methylation products in our experiments suggest that betaine has another function in cancer cells. Also, sarcosine was unlabeled, consistent with lack of choline degradation along this pathway (Fig. S1). While fresh medium did not contain betaine, the tumor-derived cells released ^13^C betaine into the medium (Fig. 3a). In HMEC cells, unlabeled betaine also appeared in the spent medium (data not shown); although the source of this betaine is not clear, it may derive from the HMEC growth supplement (a bovine pituitary extract that provides growth-stimulating hormones). Betaine can act as an osmolyte in certain cell types (Garcia-Perez and Burg 2017); however, we measured cytosolic betaine concentrations around 10–12 µM (Fig. 3b), which is far too low to affect osmotic pressure.

**Fig. 4:**
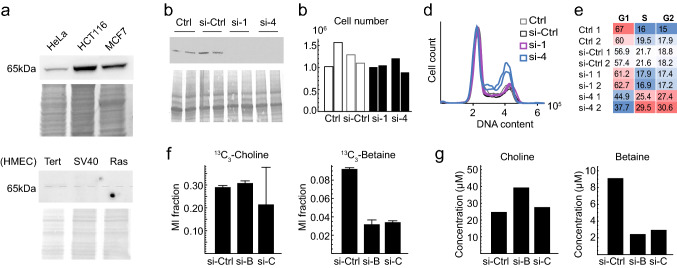
**a** CHDH expression in selected cell lines. **b**–**e** (**b**) Protein expression, **c** cell number, **d** cell cycle distribution, **e** fraction of cell cycle phases in HCT116 cells upon treatment with siRNA for CHDH. **f** MI fractions of ^13^C_3_-choline and ^13^C_3_-betaine in HeLa cells treated with siRNA for CHDH. **g** Concentrations of choline and betaine in HeLa cells treated with siRNA for CHDH

The initial enzyme in the betaine-forming choline degradation pathway (Fig. 1a) is choline dehydrogenase (CHDH). Western blotting showed that CHDH protein was indeed present in the three betaine-synthesizing cell lines, but absent in HMECs (Fig. 3a), supporting that this enzyme is responsible for the observed betaine synthesis. Interestingly, CHDH knockdown was previously reported to cause a cell cycle phenotype in siRNA screening (Kittler et al. 2007), suggesting the possibility that betaine synthesis could be important to cancer cell proliferation. To test this hypothesis, we performed transient siRNA knockdown of the CHDH enzyme in MCF7, HCT116 and HeLa cells. We observed nearly complete loss of the CHDH protein 70 h after siRNA treatment (Fig. 4b, S2). Yet, we observed no difference in cell proliferation with CHDH knockdown (Fig. 4c, S2). We did observe a reproducible increase in the G_2_/M fraction in all three cell lines, but this occurred only with two of the siRNAs (si-B and si-4) tested (Fig. 4d, 4e, S2), despite efficient knockdown with all siRNAs. ^13^C_3_ choline tracing in knockdown cells demonstrated that there was a decrease in cellular ^13^C_3_ betaine but not ^13^C_3_ choline (Fig. 4f), indicating that flux through the CHDH enzyme is suppressed. Other metabolites like sn-glyceraldehyde-3-phosphate, phosphocholine and CDP-choline, which acquired label from choline, were not affected (data not shown). We also observed lower intracellular concentration of betaine but not choline (Fig. 4g), supporting the interpretation of lower flux through the CHDH pathway. Hence, the observed cell cycle phenotype may not be related to the enzymatic function of the CHDH protein. Nevertheless, expression of CHDH coinciding with betaine synthesis in transformed cells may indicate a previously unknown fate of choline in cancer cells.

## Discussion

This study catalogues a number of metabolic products of ^13^C_3_-choline in proliferating normal and transformed cells, providing a starting point for investigating choline metabolism in depth. Some limitations are important to mention. First, we have used a single analysis protocol involving methanol extraction and HILIC chromatography, which recovers only polar metabolites; hence, our data mostly reveals soluble metabolites rather than the large variety of choline-containing lipids. Second, our metabolite annotations are tentative, based mostly on accurate mass combined with ^13^C isotope patterns, and in many cases requires further validation. It is also likely that some of the LC-HRMS peaks reported in the provided data set represent in-source fragments or other artefacts. Nevertheless, a fair number of LC-HRMS peaks are likely to represent actual cellular metabolites, as shown by the examples we have highlighted.

Besides a variety of ^13^C_3_ products containing the choline head group, our data spotlights a number of compounds with clear ^13^C_1_ and ^13^C_2_ mass isotopomers, indicating further metabolism of choline. Our finding that ^13^C_1_ mono- and ^13^C_2_ dimethyl phosphoethanolamine are derived from ^13^C_3_-choline, with comparable MI fractions, suggests a pathway where demethylation of phosphocholine forms these compounds. Reversal of the PEMT enzyme might generate these mass isotopomers, but PEMT is considered to be active only with phosphatidylcholine. A related enzyme acting on phosphocholine has been described in *C. elegans* (Brendza et al. 2007), but to our knowledge this enzyme has not been detected in mammals. Our data might indicate a previously undescribed route of phosphoethanolamine (EA-P) synthesis in human cells, but the final step of EA-P formation is not observable in our experiments, since all ^13^C methyl groups of the ^13^C_3_ choline tracer would then be lost. Future studies using 1,2-^13^C_2_-choline will be valuable to directly assess if ethanolamine formation occurs from choline. To date, the only known route of EA synthesis in mammals is that from serine, involving decarboxylation of the serine moiety of Ptd-serine to form Ptd-EA, from which EA can then be liberated by a series of lipases (Borlcenhagen, Kennedy, and Fielding 1961). We observed that only ~ 50% of P-EA derives from serine in HeLa cells (data not shown), which might indicate another source, but could also occur if the EA pools have not reached steady state.

Our finding that ^13^C_3_-phosphocholine and ^13^C_3_-sn-glycero-3-phosphocholine appears in spent medium (conditioned by cells) was unexpected. The phosphocholine zwitterion is unlikely to cross cell membranes, and to our knowledge, no phosphocholine transporter has been described in mammals. While phosphocholine is low in plasma, both phosphocholine and sn-glycero-3-phosphocholine have been found in large quantities in milk (Holmes-McNary et al. 1996; Ilcol et al. 2005), suggesting that mammary cells can indeed secrete these compounds. However, we cannot entirely exclude the possibility that the observed ^13^C_3_-phosphocholine derives from the cytosol of cells that die during the isotope tracing experiment, rather than by secretion. Interestingly, choline kinase is well known to be overexpressed in cancer cells (Glunde and Bhujwalla 2007) and high phosphocholine content has been observed in tumors (Aboagye and Bhujwalla 1999). If phosphocholine is indeed released by cancer cells, it might be detectable in serum and would be of interest as a potential tumor biomarker.

The observation that betaine is formed in tumor-derived cells, likely via the CHDH enzyme, raises questions about the function of betaine in these cells. Betaine was not used for methionine synthesis by BHMT in these cells, ruling out mechanisms dependent on methylation. Also, since intracellular concentrations were on the order of 10 µM, betaine is unlikely to function as an osmolyte as reported in other cell types (Lever and Slow 2010). Betaine is also an intermediate in the choline degradation pathway, in which dimethylglycine and sarcosine are intermediates, ultimately forming glycine. While sarcosine formation may be associated with some tumor types (Sreekumar et al. 2009), we did not observe formation of these products from betaine. Other studies indicate that betaine may enhance antioxidant responses in cells (Zhang et al. 2016), and this potential function of betaine merits further investigation. Finally, our knockdown studies of CHDH indicate that suppression of betaine synthesis does not give any noticeable growth phenotype in standard culture conditions; however, this does not rule out a possible role for betaine in conditions closer to the tumor environment, which could be tested for example in hypoxia or in 3D culture models. Additional work is needed to address in more depth the role of betaine synthesis in transformed cells.

## Electronic supplementary material

Below is the link to the electronic supplementary material.

Electronic supplementary material 1 (PDF 133 kb)

Electronic supplementary material 2 (XLSX 169 kb)
