# Supplementary material for: Mapping choline metabolites in normal and transformed cells
Source: Metabolomics. 2020 Nov 29;16(12):125. doi: 10.1007/s11306-020-01749-0 (PMC7701132; doi:10.1007/s11306-020-01749-0)

Supplementary material for:

Mapping choline metabolites in normal and transformed cells

Irena Roci[1,2,3], Jeramie D. Watrous[4], Kim A. Lagerborg[4], Mohit Jain[4], and Roland Nilsson[1,2,3] *

[1] Cardiovascular Medicine Unit, Department of Medicine, Karolinska Institutet, SE-171 76 Stockholm, Sweden.
[2] Division of Cardiovascular Medicine, Karolinska University Hospital, SE-171 76 Stockholm, Sweden.
[3] Center for Molecular Medicine, Karolinska Institutet, SE-171 76 Stockholm, Sweden.
[4] University of California, San Diego, Department of Medicine & Pharmacology, 9500 Gilman Avenue, La Jolla, California 92093, United States.

[*] Correspondence: roland.nilsson@ki.se; Tel.: +46 722 334 580

SUPPLEMENTARY FIGURES

Fig. S1. M+0 and M+1 mass isotope fractions of methionine, sarcosine and 1-methylnicotinamide in cell extracts and spent media of the selected cell lines.

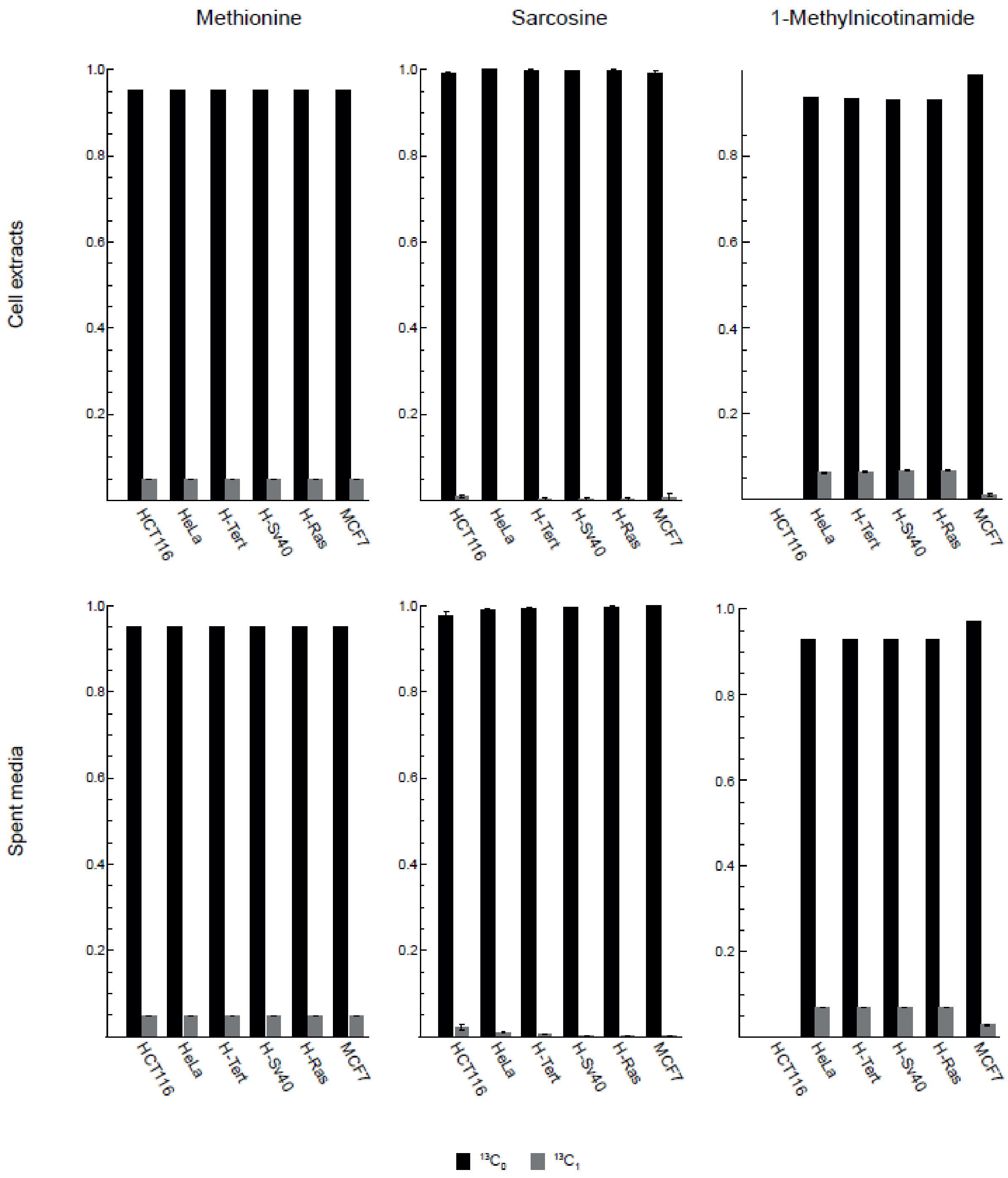

Fig. S2. a-d a) Protein expression, C) Cell number, D) Cell cycle distribution, E) Fraction of cell cycle phases in MCF7 and HeLa cells upon treatment with siRNA for CHDH.

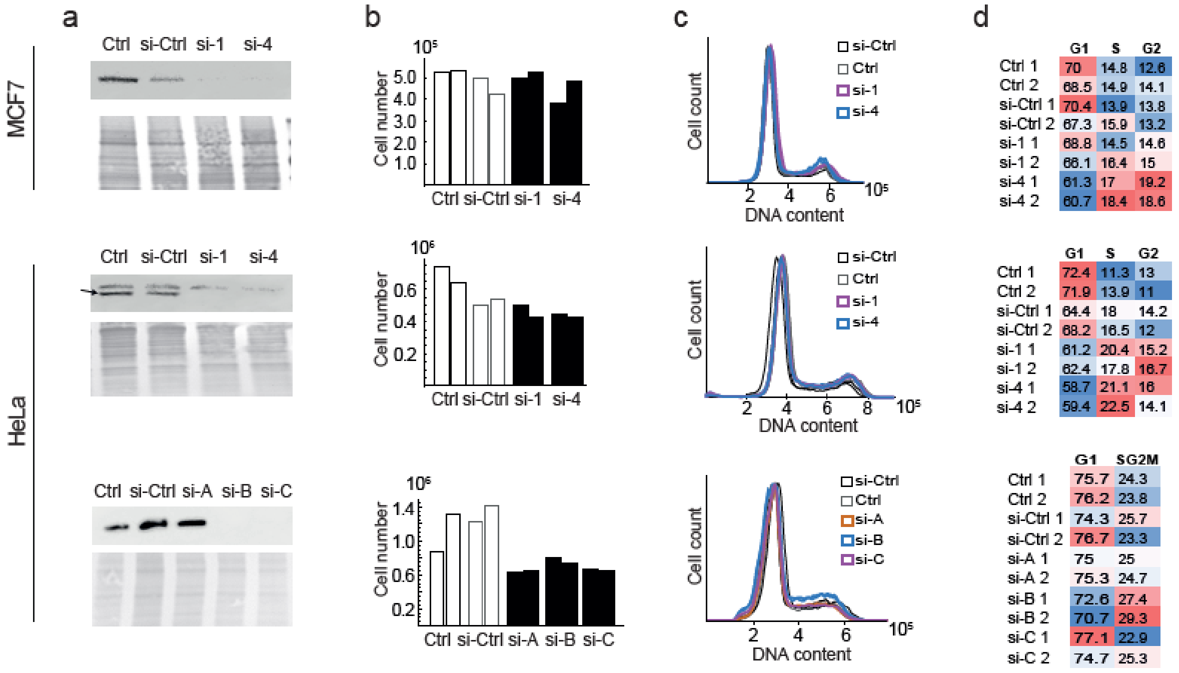

Supplement: Supplementary file 1 — Electronic supplementary material 1 (PDF 133 kb) [file 11306_2020_1749_MOESM1_ESM.pdf]
